# *Callitetrarhynchus gracilis* (Rudolphi, 1819) Pintner, 1931 (Cestoda: Trypanorhyncha) parasitizing the musculature of *Sardinella brasiliensis* (Steindachner, 1879) (Actinopterygii) off the coast of the state of Rio de Janeiro, Brazil

**DOI:** 10.1371/journal.pone.0206377

**Published:** 2018-11-14

**Authors:** Priscila Queiroz Faria de Menezes, Marcelo Knoff, Nilza Nunes Felizardo, Nathalie Costa da Cunha, Erich Loza Telleria, Eduardo José Lopes Torres, Laura Costa Borges, Elmiro Rosendo do Nascimento, Sergio Carmona de São Clemente

**Affiliations:** 1 Laboratório de Inspeção e Tecnologia de Pescado, Universidade Federal Fluminense, Niterói, RJ, Brazil; 2 Laboratório de Helmintos Parasitos de Vertebrados, Instituto Oswaldo Cruz, Fiocruz, Rio de Janeiro, RJ, Brazil; 3 Laboratório de Epidemiologia Molecular, Departamento de Saúde Coletiva Veterinária e Saúde Pública, Universidade Federal Fluminense, Niterói, RJ, Brazil; 4 Laboratório de Biologia Molecular de Parasitas e Vetores, Instituto Oswaldo Cruz, Fiocruz, Rio de Janeiro, RJ, Brazil; 5 Laboratório de Helmintologia Romero Lascasas Porto, Departamento de Microbiologia, Imunologia e Parasitologia, Universidade Estadual do Rio de Janeiro, Rio de Janeiro, RJ, Brazil; Animal Health Centre, CANADA

## Abstract

Cestodes of the order Trypanorhyncha can frequently be found infecting the muscles of several marine fish species, and lead to a repugnant aspect of the fish and rejection by consumers. The Brazilian sardinella, *Sardinella brasiliensis*, occurs from the Gulf of Mexico and the Caribbean to northern Uruguay. In southeastern Brazil, fishing for the species is very intensive since it generates significant revenue as one of the most commonly consumed fish and an important element of the canned fish industry. The aims of the present study were to identify and report the occurrence of tiny cestodes (3 mm—6.6 mm) in the musculature of Brazilian sardinella that were purchased in the São Pedro fish market in the municipality of Niterói, state of Rio de Janeiro, Brazil. From October 2013 to November 2016, 75 specimens of *S*. *brasiliensis* were investigated. The trypanorhynch cestodes encountered were identified as plerocerci of *Callitetrarhynchus gracilis* using morphological, morphometric and molecular data. Parasitic indices were calculated, and the cestodes infection of the musculature of Brazilian sardinella had the following values: prevalence, 40%; mean intensity, 3.47; mean abundance, 1.39; and range of infection, 1–18, specimens. The presence of this trypanorhynch cestode in the musculature of Brazilian sardinella is an important indicator of fish hygiene.

## Introduction

The Brazilian sardinella, *Sardinella brasiliensis* (Steindachner, 1879), occurs from the Gulf of Mexico and the Caribbean to northern Uruguay. In southeastern Brazil, the fishery for the species is very intense since it is one of fish most frequently consumed by the population and is an important element of the canned fish industry, thus generating significant income throught its exportation. The species feeds mainly on planktonic organisms filtered by gill rakers [1].

In terms of volume, Brazilian sardinella is the most important marine fishery resource in Brazil, with landings concentrated in the states of Rio de Janeiro, São Paulo and Santa Catarina. The species contributes to world food resources in two ways: directly, through actual consumption (fresh, frozen or processed) and indirectly, by providing products used for animal feeds and fertilizers or by serving as bait to catch other fishes [2,3].

According to the Food and Agriculture Organization of the United Nations (FAO), among the 23 fish species and genera most frequently captured worldwide in 2011 and 2012, *Sardinella* spp. stood out in fourth place [4].

Cestodes of the order Trypanorhyncha are of hygienic importance because of their repugnant aspect, particularly when teleostean fish present massive infections in their musculature and organs that can make commercialization infeasible due to sanitary inspection and/or rejected by the consumer [5,6,7]. The order Trypanorhyncha possesses a great diversity of species with global distributions. As adults, these worms inhabit the stomach and intestine of elasmobranch fish, while the larval forms can infect a great diversity of teleostean fish and marine invertebrates in tropical and subtropical regions, but rarely freshwater fish and other vertebrates [8,9]. These cestodes cause significant economic losses. Furthermore, accidental infections of humans by larval trypanorhynchs have been reported due to the ingestion of raw fish meat. Although these infections do not present zoonotic potential, recent research reports that these cestodes can cause allergic disorders in humans since immunological hypersensitivity has been demonstrated for some species in studies using a murine model [10,11,12,13].

In Brazil, *S*. *brasiliensis* was reported being parasitized by trypanorhynch cestodes of the species *Nybelinia sp*. and *Callitetrarhynchus gracilis* (Rudolphi, 1819) Pintner, 1931 in the body cavity [14].

The aims of the present study were to identify, to species level, and report the occurrence of tiny cestode specimens parasitizing the musculature of the Brazilian sardinella that were purchased in the São Pedro fish market in the municipality of Niterói, state of Rio de Janeiro, Brazil.

## Materials and methods

### Collection and examination of fish for cestode

From October 2013 to November 2016, 75 specimens of *S*. *brasiliensis*, total length 18–23 (20.2) cm, were acquired from the São Pedro fish market in the municipality of Niterói, state of Rio de Janeiro, Brazil (22° 53' 00" S; 43º 06' 13" W). The specimens were transported in isothermal containers with ice to the Laboratório de Inspeção e Tecnologia de Pescado da Faculdade de Veterinária, Universidade Federal Fluminense (Fishery Inspection and Technology Laboratory of the Fluminense Federal University), for necropsy [15], where they were identified according to Figueredo and Menezes [1] and Froese and Pauly [16]. After necropsy, musculature was transferred to Petri dishes containing physiological solution with 0.65% NaCl. Cestode blastocysts were removed from the musculature for further investigation. The plerocerci were removed from the blastocysts with the aid of sharp needles under a stereomicroscope to release the larvae, which were put in the refrigerator in Petri dishes with distilled water for at least 24 h to permit relaxation of the scolex and tentacular extroversion. Some of the specimens were fixed in AFA [70% ethanol (93 parts), formalin (5 parts) and glacial acetic acid (2 parts)] and preserved in 70% ethanol. The cestodes were collected, fixed, and preserved in accordance with Knoff and Gomes [15].

### Morphological description

Ten plerocerci of the trypanorhynchs were stained with Langeron´s carmine, cleared in beechwood creosote, and preserved as whole mounts on Canada balsam according to Knoff and Gomes [16]. The classification of Trypanorhyncha followed Palm [9]. For the identification of trypanorhynch cestode larvae, Dollfus [17], Carvajal and Rego [18], São Clemente [19] and Palm [9] were used. The terminology for cestode microtriches followed Chervy [20]. Measurements were obtained by bright field microscopy using an Olympus BX 41 microscope. Drawings were made using a drawing tube connected to the microscope. The samples were then analyzed by bright-field microscopy with a Zeiss Axiophot microscope using a Nomarski’s differential interference contrast (DIC) apparatus, with images obtained with a Canon digital camera (Power Shot A640). Six specimens were prepared for scanning electron microscopy (SEM) as described by Lopes Torres et al. [21]. The samples fixed in 70% ethanol were dehydrated in an ethanol series (70% to 100%), CO2 critical point dried, coated in gold, and then examined and photographed using a SEM (Jeol JSM-6390LV), under 15 kV acceleration voltage. Measurements are provided in millimeters (mm) with averages and standard deviations in parentheses, unless otherwise indicated.

### DNA extraction, PCR amplification and sequencing

For genetic analysis, ten specimens of *C*. *gracilis* from seven different fish were investigated. The specimens were placed in phosphate-buffered saline (PBS) and frozen individually in microtubes at -20°C. Samples were then deposited in liquid nitrogen and crushed with a sterile micropestle followed by DNA extraction using a MasterPure DNA purification kit (Epicentre, Madison, Wisconsin, USA). The DNA was amplified using the Sigma Genosys taxon-specific polymerase chain reaction (PCR) with 28S rRNA gene primers ZX-1 (5'-CCCGCTGAATTTA AGCATAT-3') and 1500R (5'-GCTATCCTGAGGGAAACTTCG-3'), as modified by Van der Auwera et al. [22], and 18S rRNA gene primers WormA (5'-GCGAATGGCTCATTAAATCAG-3') and WormB (5'-CTTGTTACGACTTTTACTTCC-3') [23]. PCR was carried out in a final volume of 50 μl containing a mixture of 2 μl of DNA isolate, 1.25 U of Taq polymerase (Platinum Taq DNA Polymerase, Thermo Fisher Scientific, Waltham, Massachusetts, USA), 1× PCR buffer (10mM of Tris–HCl, pH 8.0; and 50mM of KCl), 2- mM of MgCl2, 0.2 mM of deoxynucleoside triphosphate (dNTP) mixture and 0.2mM of forward and reverse primers. A negative control (ultrapure water) was included in the PCR reaction. The amplification of parasite DNA was performed using a MyCycler thermocycler (Thermo, Foster City, California, USA), following the cycle of the primers, Worm A and Worm B, according to Littlewood and Olson [23]. The procedure comprised an initial denaturation step at 94°C for 2 min, followed by 15 cycles of denaturation at 94°C for 30 s, primer annealing at 54°C for 30 s and extension at 72°C for 30 s. This was followed by 45 cycles of 30 s at 94°C (denaturation), 30 s at 54°C (annealing) and 30 s at 72°C, with an additional final elongation for 10 min at 72°C. The PCR products were stained using GelRed and were viewed by means of electrophoresis on a 1.5% agarose gel. Amplicons of expected size were purified by means of the Wizard SV Gel and PCR Clean-Up kit (Promega, Madison, Wisconsin, USA). After screening, 28SrRNA gene PCR products were sequenced with the primers used in PCR and internal primers 300F (5´-CAAGTACCGTGAGGG AAAGTTG-3´), ECD2 (5´-CTTGGTCCGTGTTTCAAGACGGG-3´), 400R (5´-GCA GCTTG ACTACACCCG-3´) and 1090F (5´-TGAAACACGGACCAAGG-3´). The 18SrRNA gene PCR products were sequenced with the primers used in PCR and internal primers 300F (5´-AGGGTTCGATTCCGG AG-3´), 600R (5´-ACCGCGGCKGCTGGCACC-3´), 930F (5´-GCATGGAATAATGGAATAGG-3´), 1200F (5´-C AGGTCTGTGATGCCC-3´) and 1200F (5´-C AGGTCTGTGATGCCC-3´) [24]. Forward and reverse nucleotide sequences were determined using a DNA sequence analyzer (ABI3730xlv; Thermo Fisher Scientific, Waltham, Massachusetts, USA).

Partial *C*. *gracilis* 18S or 28S rRNA gene sequences (RJ isolate) were trimmed for quality and assembled in contigs with CLC Main Workbench software, version 7.6.4 (Qiagen, Aarhus A/S, Denmark). The resulting consensus sequences were used as query for nucleotide BLAST analysis [25] against the NCBI database in order to determine similarity to species of the trypanorhynch family Lacistorhynchidae. Closely related 18S or 28S rRNA gene sequences from other organisms were selected from GenBank [26] and were used in multiple alignments together with sequences from *Prochristianella clarkeae*, *Ditrachybothridium macrocephalum* and *Echinobothrium harfordi* chosen as outgroups. Regions that could not be unambiguously aligned due to length variation were excluded from the analysis. All sequence analyses and multiple alignments were performed using the CLC Main Workbench software. A phylogram was generated using Mega6 software, version 6.06 [27] with 18S and 28S rRNA sequences concatenated in one single alignment and the neighbor-joining method with bootstrapping of 10,000 replications.

### Parasitic indices

The parasitic indices used were those described by Bush et al. [28], and abbreviated as: P = prevalence, MI = mean intensity, MA = mean abundance and RI = range of infection. Voucher specimens were deposited in the Helminthological Collection of the Oswaldo Cruz Institute (CHIOC), FIOCRUZ, Rio de Janeiro, RJ, Brazil.

## Results

### Morphological identification

The analysis of 75 necropsied specimens of *S*. *brasiliensis* revealed 30 infected fish with a total of 104 plerocerci, of which 91 were in abdominal musculature, 12 in dorsal musculature and 1 in caudal musculature. Parasitic indices were 40% of prevalence; 3.47 of mean intensity; 1.39 of mean abundance; and with range of infection of 1–18 specimens. The voucher specimens were deposited in the CHIOC under the numbers: 38887, 38888, 38889, 38890, 38891, 38892, 38893a-b, 38894. Trypanorhynch plerocerci were taxonomically identified as below.

Lacistorhynchoidea Guiart, 1927

Lacistorhynchidae Guiart, 1927

Lacistorhynchinae Guiart, 1927

*Callitetrarhynchus* Pintner, 1931

**Fig 1:**
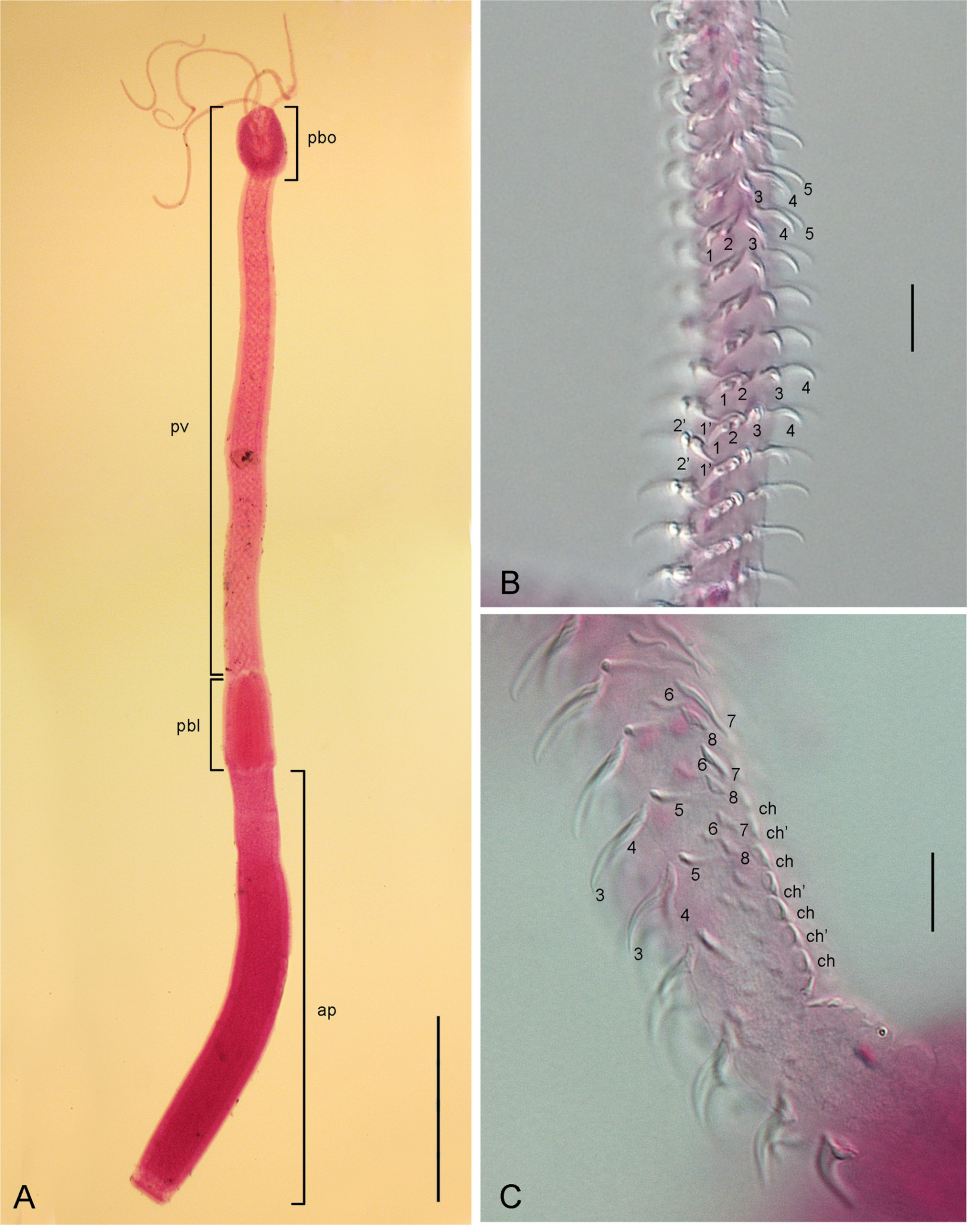
*Callitetrarhynchus gracilis* plerocercus from *Sardinella brasiliensis*. (A) Entire plerocercus, pars bothrialis (pbo), pars vaginalis (pv), pars bulbosa (pbl) and appendix (ap). Bar = 400 μm. (B) Detail of the arrangement of the hooks of the internal surface of the metabasal armature tentacle; shown are hooks 1(1’), 2 (2'), 3, 4 and 5. Bar = 25 μm. (C) Detail of the arrangement of the hooks of the external surface of the basal armature tentacle; shown are hooks 3, 4, 5, 6, 7 and 8 and the chainette (ch and ch’). Bar = 10 μm.

**Fig 2:**
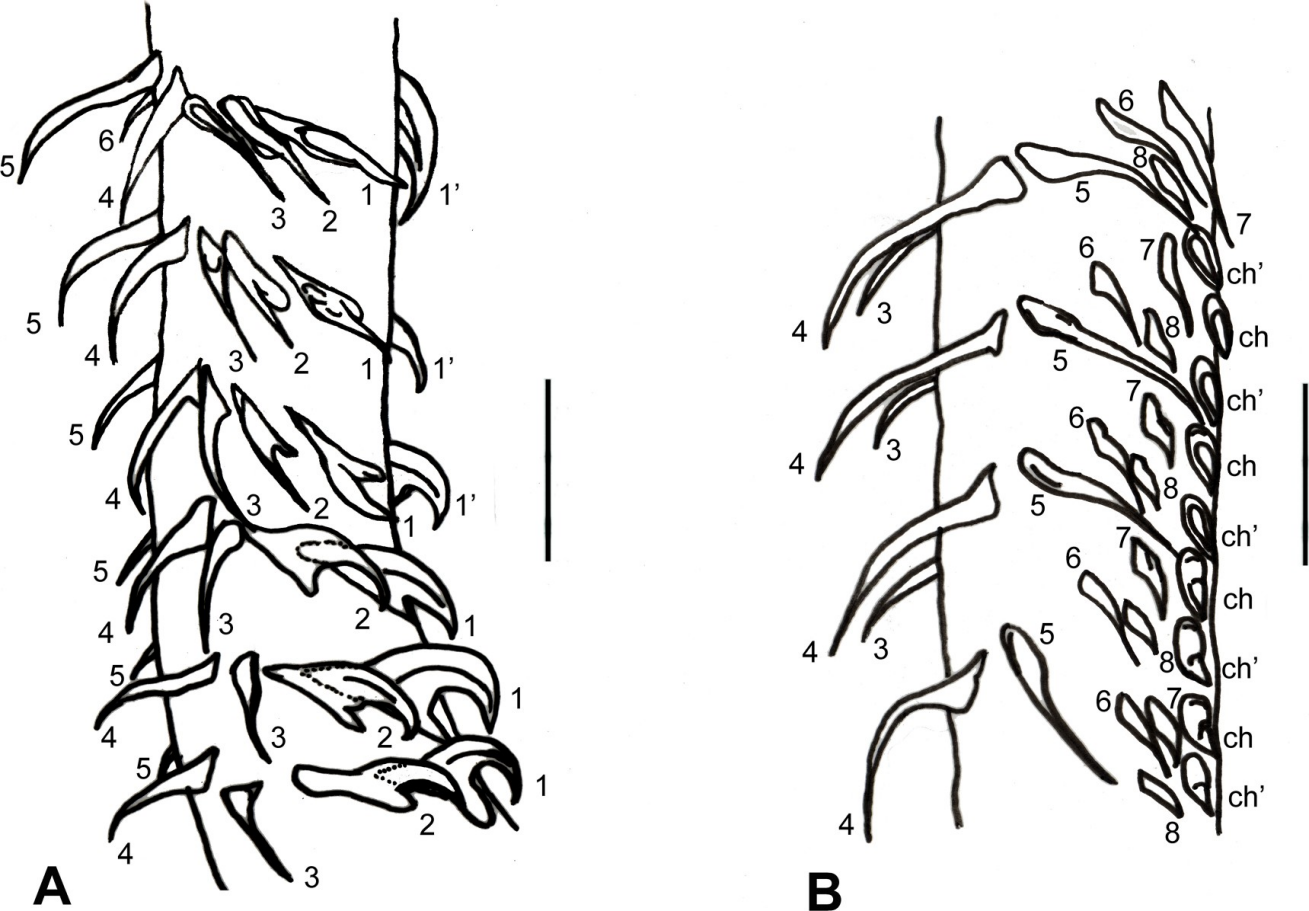
*Callitetrarhynchus gracilis* plerocercus from *Sardinella brasiliensis*. (A) Detail of the arrangement of the hooks of the antibothrial surface of the metabasal armature tentacle; shown are hooks 1(1’), 2, 3, 4, 5 and 6. Bar = 10 μm. (B) Detail of the arrangement of the hooks of the antibothrial surface of the metabasal armature tentacle; shown are hooks 3, 4, 5, 6, 7 and 8 and the chainette (ch and ch’). Bar = 10 μm.

**Fig 3:**
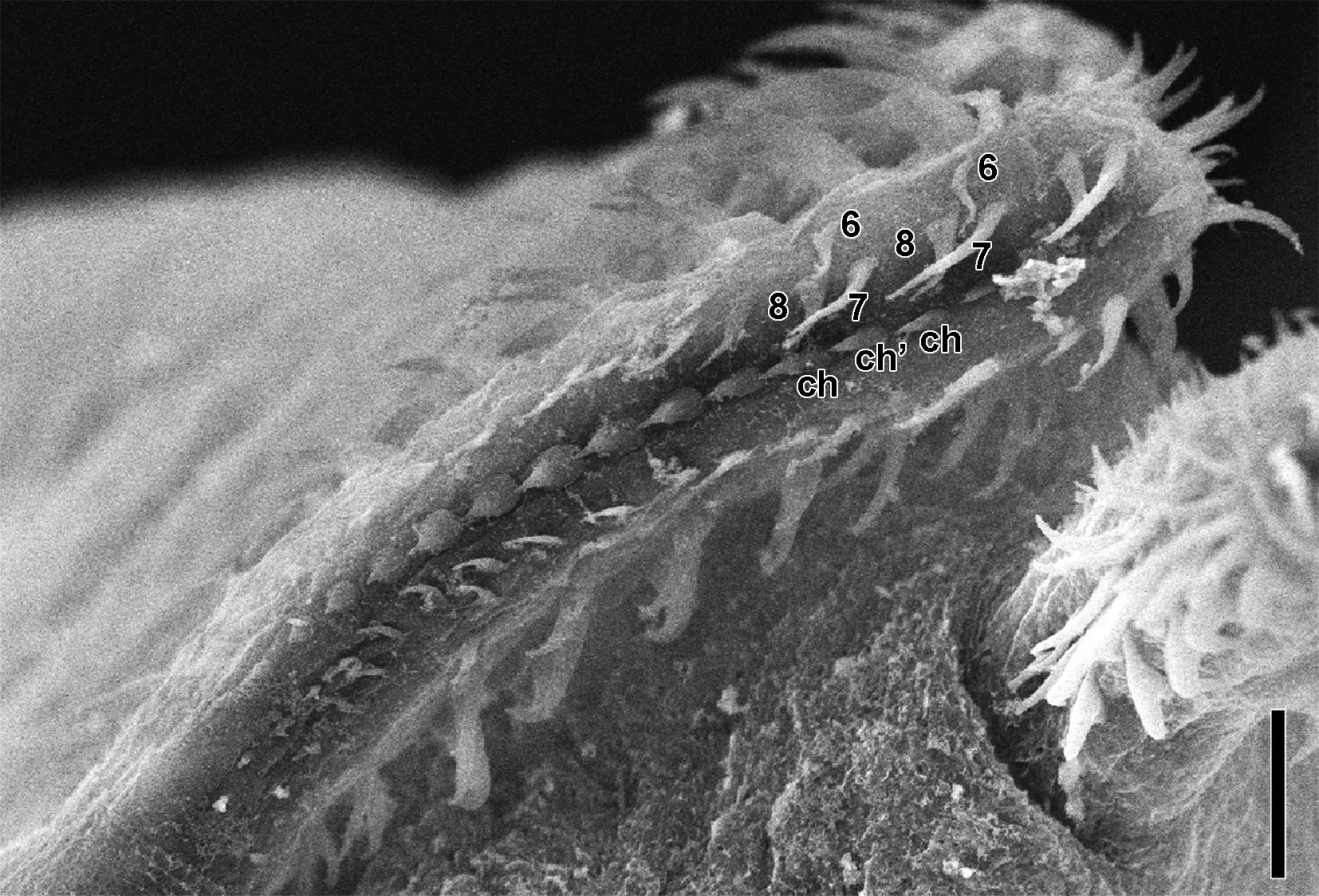
*Callitetrarhynchus gracilis* plerocercus from *Sardinella brasiliensis*, SEM. Detail of the arrangement of the hooks of the external surface of the basal armature tentacle; shown are hooks 6, 7 and 8 and the chainette (ch and ch’). Bar = 10 μm.

**Fig 4:**
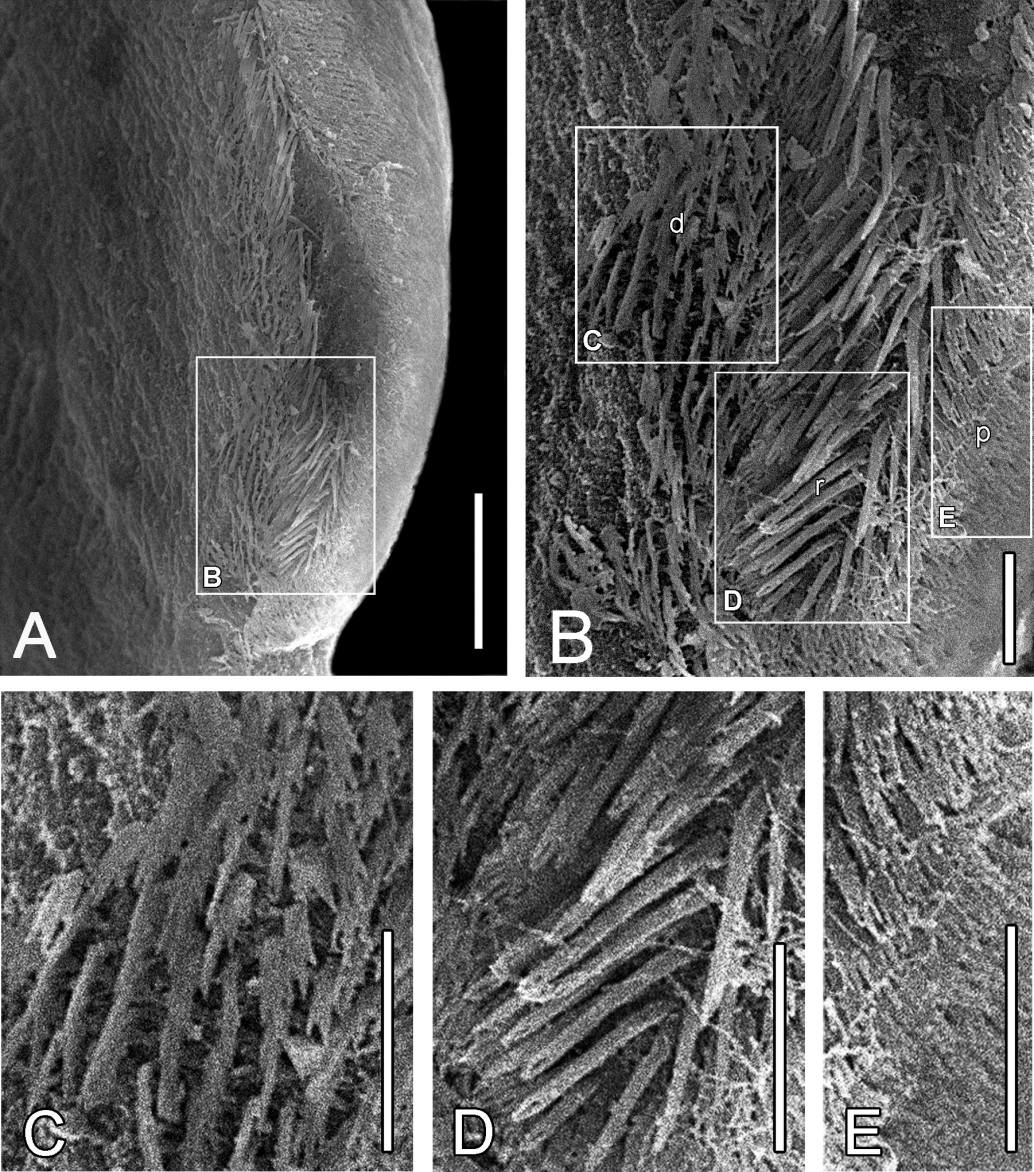
*Callitetrarhynchus gracilis* plerocercus from *Sardinella brasiliensis*, SEM. (A) Bothrial rim, showing the microtriches on the transition zone between proximal and distal surfaces. Rectangle B indicates the surface where the figure was obtained. Bar = 20 μm. (B) Detail of the bothrial surface showing the different sizes and lengths of microtriches on the bothrial rim (r) and on the adjacent distal (d) and proximal (p) bothrial surfaces. Rectangles C, D and E indicate the surfaces where the figures were obtained. Bar = 5 μm. (C) Distal bothrial surface, trifid spinitriches and capilliform filitriches. Bar = 5 μm. (D) Bothrial rim surface, chelate spinitriches and capilliform filitriches. (E) Proximal bothrial surface, trifid spinitriches and capilliform filitriches. Bar = 5 μm.

*Callitetrarhynchus gracilis* (Rudolphi, 1819) Pintner, 1931 (Figs 1–4)

Description of the morphological features was based on 16 specimens. Plerocerci with blastocyst and caudal extension. Scolex elongated with appendix, thin and acraspedote. Two patelliform bothria with weakly notched posterior margins. Pars vaginalis long, tentacle sheaths regularly sinuous, less sinuous in the pars bothrialis region. Bulbs elongated. Retractor muscles originate in anterior 1/3 of bulbs. Pars postbulbosa present, small, absent in some. Metabasal armature poeciloacanthous atypical, heteromorphous; hooks hollow, in ascending half spirals of 8 principal hooks, beginning on internal surface. Hooks 1(1´) are large and uncinate; hooks 2(2´) are uncinate and long; hooks 3(3´) are falciform, large and have large bases; hooks 4(4´) and 5(5´) are falciform; hooks 6(6´) are spiniform and located near the external surface; the satellite hooks 7(7') are larger than hooks 8(8'), both of which are slender, uncinate. A simple chainette is present. By SEM, it was possible observe the bothrial rim with microtriches on the transition zone between proximal and distal surfaces. Bothrial surfaces showed distinct sizes and lengths of microtriches on the bothrial rim and on the adjacent distal- and proximal bothrial surfaces; trifid spinitriches and capilliform filitriches present on the distal bothrial surface; chelate spinitriches and capilliform filitriches present on the bothrial rim surface; trifid spinitriches and capilliform filitriches present on the proximal bothrial surface.

The morphological and morphometric data for 10 *C*. *gracilis* plerocerci collected from *S*. *brasiliensis* are presented in Table 1.

**Table 1:** Morphological and morphometric data for *Callitetrarhynchus gracilis* plerocerci collected from *Sardinella brasiliensis* off the coast of the state of Rio de Janeiro, Brazil.

*Callitetrarhynchus gracilis*	
Scolex (L)	1.8–3.5 (2.8±0.44)
Scolex (W)	0.1–0.3 (0.2±0.05)
Appendix (L)	0.9–3.1 (1.9±0.61)
Appendix (W)	0.1–0.2 (0.2±0.04)
Pars bothrialis (L)	0.2–0,3 (0.2±0.03)
Pars bothrialis (W)	0.1–0.2 (0.2±0.03)
Pars vaginalis (L)	1.3–2.9 (2.2±0.41)
Pars bulbosa (L)	0.1–0.5 (0.3±0.1)
Pars bulbosa (W)	0.1–0.4 (0.2±0.08)
Pars post-bulbosa (L)	0–0.05
Bulbs (L)	0.3–0.5 (0.4±0.05)
Bulbs (W)	0.05–0.09 (0.06±0.01)

L = length; W = width. Measurements are presented in millimeters.

### Molecular analyses

**Fig 5:**
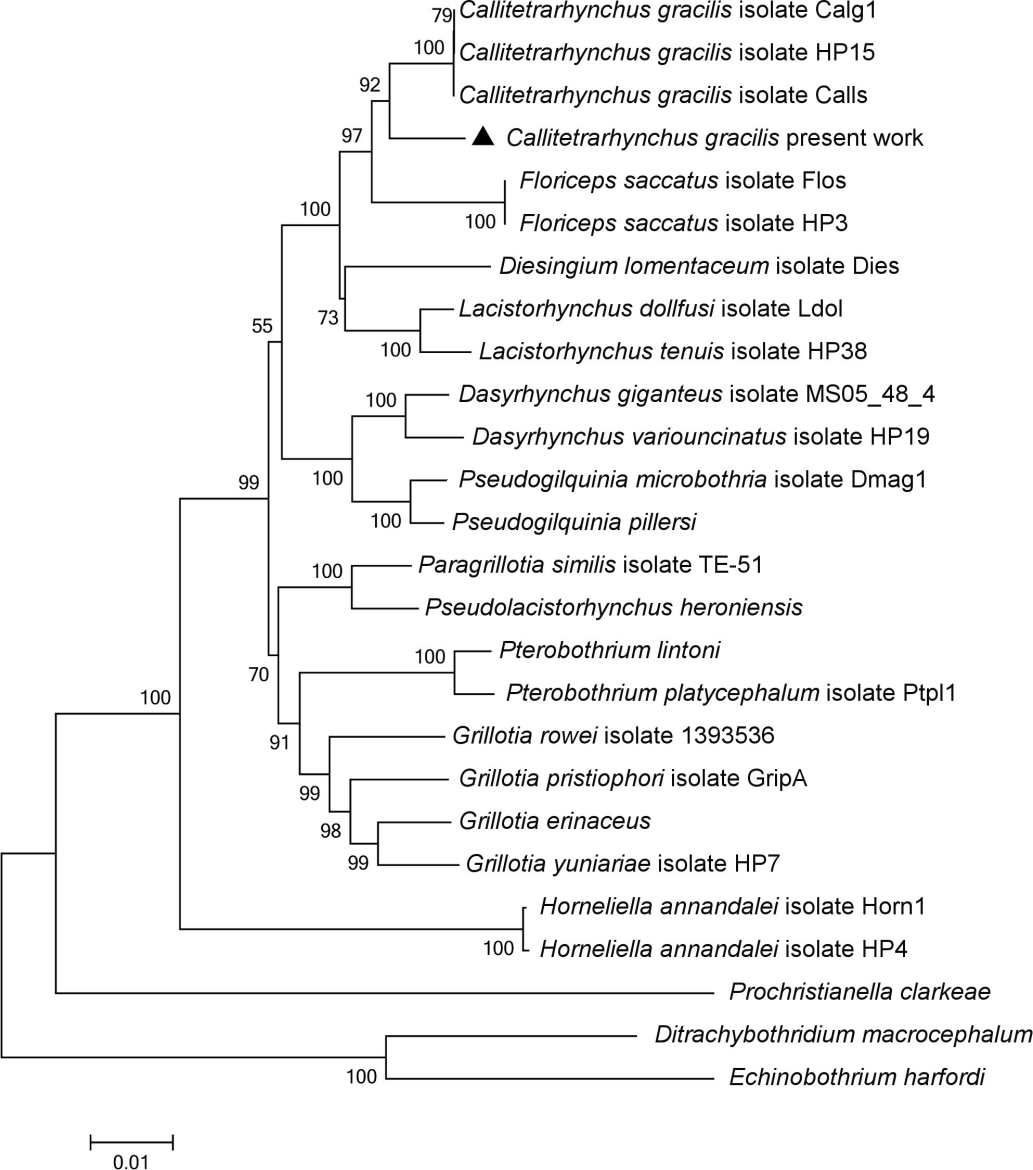
Neighbor-joining phylogram inferred from concatenated partial DNA sequences of *Callitetrarhynchus gracilis*. Branches for each DNA sequence with species names are indicated, corresponding GenBank accession numbers are listed on Table 2; bootstrap values are indicated on nodes. Scale bar represents the expected number of substitutions per nucleotide.

Ten specimens were used for partial sequences from 18S rRNA (1932 nucleotides, GenBank MG693781) and 28S rRNA (1452 nucleotides, GenBank MG694210) were obtained in the present study. Comparison of this sequence with other sequences of the same gene available in the GenBank database showed that the 18S rRNA and 28S rRNA sequences share 98% and 99% similarity, respectively, with other *Callitetrarhynchus* 18SrRNA (DQ642920, DQ642921, and FJ572921) and 28SrRNA (DQ642758, DQ642759, and FJ572957) sequences from isolates Calg1, Calls and HP15 available on NCBI GenBank (Table 2). The phylogram generated (Fig 5) using sequences from *C*. *gracilis* and other species of Lacistorhynchidae shows that the *C*. *gracilis* RJ isolate (present work) groups with other *C*. *gracilis* isolates.

**Table 2:** Sequences used for phylogenetic analysis.

Species name	18S ribosomal RNA gene accession number	Identity with *C*. *gracilis* isolate RJ	28S ribosomal RNA gene accession number	Identity with *C*. *gracilis* isolate RJ
*Callitetrarhynchus gracilis* isolate Calg1	DQ642920	98%	DQ642758	99%
*Callitetrarhynchus gracilis* isolate Calls *	DQ642921	98%	DQ642759	98%
*Callitetrarhynchus gracilis* isolate HP15	FJ572921	98%	FJ572957	99%
*Callitetrarhynchus gracilis* isolate RJ (present work)	MG693781	n/a	MG694210	n/a
*Floriceps saccatus* isolate Flos	DQ642919	98%	DQ642757	96%
*Floriceps saccatus* isolate HP3	FJ572922	98%	FJ572958	96%
*Diesingium lomentaceum* isolate Dies	DQ642922	97%	DQ642760	96%
*Lacistorhynchus dollfusi* isolate Ldol	DQ642923	97%	DQ642761	97%
*Lacistorhynchus tenuis* isolate HP38	FJ572919	96%	FJ572955	97%
*Dasyrhynchus giganteus* isolate MS05_48_4	FJ788112	96%	FJ788109	95%
*Dasyrhynchus variouncinatus* isolate HP19	FJ572914	96%	FJ572950	95%
*Pseudogilquinia microbothria* isolate Dmag1	DQ642928	96%	DQ642766	95%
*Pseudogilquinia pillersi*	AJ287496	96%	AF286964	94%
*Paragrillotia similis* isolate TE-51	KF685779	97%	KF685909	95%
*Pseudolacistorhynchus heroniensis*	AJ287519	96%	AF286968	95%
*Pterobothrium lintoni*	AF287004	96%	AF286973	93%
*Pterobothrium platycephalum* isolate Ptpl1	DQ642926	96%	DQ642764	94%
*Grillotia rowei* isolate 1393536	DQ642927	96%	DQ642765	94%
*Grillotia pristiophori* isolate GripA	DQ642925	96%	DQ642763	95%
*Grillotia erinaceus*	AJ228781	96%	AF286967	95%
*Grillotia yuniariae* isolate HP7	FJ572916	96%	FJ572952	95%
*Hornelliella annandalei* isolate Horn1	DQ642924	95%	DQ642762	88%
*Hornelliella annandalei* isolate HP4	FJ572920	95%	FJ572956	89%
*Prochristianella clarkeae*	DQ642947	92%	KX086307	84%
*Ditrachybothridium macrocephalum*	DQ642903	91%	KR653220	83%
*Echinobothrium harfordi*	AF286985	90%	AF286921	82%

* named according to Palm *et al*. [24]

## Discussion

The specimens of *C*. *gracilis* in the present study possess morphological characteristics that are in accordance with the morphological data of the redescriptions of Dollfus [17], Carvajal and Rego [18], São Clemente [19] and Palm [9]. Several occurrences of *C*. *gracilis* in marine fish in Brazil and in the world have recently been reported [29,30,31,32,33]. The morphology of microtriches observed on the bothrial rim, of the transition zone between proximal and distal surfaces are in accordance with observations of Palm [9] and Chervy [20]. The specimens of *C*. *gracilis* in the present study were similar to the small plerocerci parasitizing some teleost fish from Brazil and other countries, as indicated by Palm [9,34], who reported that this trypanorhynch species exhibited a remarkable size range within different hosts. This observation could corroborate the suggestion of Palm [9,34] that *C*. *gracilis* has a four host trophic-chain life cycle. Because, the plerocercus with small form occurs in general in smaller fish species, as cluppeids, and the large form in larger fish, as scombrids, paralichthyids, balistids, and this constancy, has been demonstrated through the hosts.

*Callitetrarhynchus gracilis* was previously reported parasitizing *S*. *brasiliensis* off the coast of the state of Rio de Janeiro by Moreira et al. [14]. However, these authors did not provide morphometric data. After analyzing the specimens collected by them deposited in CHIOC under the numbers 37936 and 37937, they were found to be small (scolex 1.67–2.52 mm; appendix 1.37–1.40) like the specimens observed in the present study.

The parasitic indices for *C*. *gracilis* in the present study were higher then those of Moreira et al. [14], who reported a prevalence of 15%, mean intensity of 1.1 ± 0.3 and mean abundance of 0.3. However, the site of infection, the body cavity, reported by Moreira et al. [14] differed from that of the present study which represents the first report of this parasite in the musculature of *S*. *brasiliensis*.

The trypanorhynch species of the present study was found to be most similar to other species within Lacistorhynchoidea. The phylogenetic analysis performed with the 18S and 28S rRNA concatenated multiple alignment supported its taxonomic position within the genus *Callitetrarhynchus*, and as the sister taxon of *Floriceps saccatus*. This finding was in agreement with Olson et al. [35]. Therefore, according to data from the morphological and molecular analyses presented here, the investigated parasite indeed belongs to subfamily Lacistorhynchinae. Additionally, morphological data strongly supports its classification as *C*. *gracilis*. Furtheremore, the presence of *C*. *gracilis* in the musculature of *S*. *brasiliensis* originating from off the coast of Rio de Janeiro, Brazil, found in the present study represents a new location of infection for this trypanorhynch. Plerocerci of *C*. *gracilis* are usually reported in the abdominal cavity from many fish species, reports in the musculature are uncommon and this may serve as a warning for future research on this species of fish from this region.

## Conclusions

The results of the PCR and sequencing effectively identified the species *C*. *gracilis*, and corroborate the morphological identification of this species.

This species of parasite was studied because of its importance during the sanitary inspection of fish. The presence of trypanorhynch cestodes in the musculature of Brazilian sardinella is important for food hygiene because parasitized fish are usually rejected by consumers due to their appearance cause of their repugnant aspect. Furthermore, accidental infections of humans by them have been reported due to the ingestion of raw fish meat. Although these infections do not present zoonotic potential, research reports that them can cause allergic disorders in humans.
